# Critical Role for the Protons in FRTL-5 Thyroid Cells: Nuclear Sphingomyelinase Induced-Damage

**DOI:** 10.3390/ijms150711555

**Published:** 2014-06-27

**Authors:** Elisabetta Albi, Giuseppina Perrella, Andrea Lazzarini, Samuela Cataldi, Remo Lazzarini, Alessandro Floridi, Francesco Saverio Ambesi-Impiombato, Francesco Curcio

**Affiliations:** 1 Laboratory of Nuclear Lipid BioPathology, Research Center of Biochemical-Specialized Analyses, Perugia 06100, Italy; E-Mails: andrylazza@gmail.com (A.L.); samuelacataldi@libero.it (S.C.); Remo30@libero.it (R.L.); direzione@crabion.it (A.F.); 2 Department of Clinical and Biological Sciences, University of Udine, Udine 33100, Italy; E-Mails: ambesis@me.com (F.S.A.-I.); curcio@uniud.it (F.C.)

**Keywords:** protons, thyroid cells, sphingomyelin, neutral-sphingomyelinase, apoptosis, sphingomyelin-synthase

## Abstract

Proliferating thyroid cells are more sensitive to UV-C radiations than quiescent cells. The effect is mediated by nuclear phosphatidylcholine and sphingomyelin metabolism. It was demonstrated that proton beams arrest cell growth and stimulate apoptosis but until now there have been no indications in the literature about their possible mechanism of action. Here we studied the effect of protons on FRTL-5 cells in culture. We showed that proton beams stimulate slightly nuclear neutral sphingomyelinase activity and inhibit nuclear sphingomyelin-synthase activity in quiescent cells whereas stimulate strongly nuclear neutral sphingomyelinase activity and do not change nuclear sphingomyelin-synthase activity in proliferating cells. The study of neutral sphingomyelinase/sphingomyelin-synthase ratio, a marker of functional state of the cells, indicated that proton beams induce FRTL-5 cells in a proapoptotic state if the cells are quiescent and in an initial apoptotic state if the cells are proliferating. The changes of cell life are accompanied by a decrease of nuclear sphingomyelin and increase of bax protein.

## 1. Introduction

Proton beams induced chromosomal aberrations [1,2]. The relative biological effectiveness was both dose- and depth-dependent [3]. In HTB63 human melanoma cells, proton beams inhibited cell growth with G2/M and G1/G0 arrest of the cell cycle and appearance of apoptotic nuclei, even 48 h after irradiation [4].

To date we have very little information on the effect of protons on the endocrine system. Just a paper exists about the thyroid, an endocrine gland that regulates the metabolism of cardiovascular, musculoskeletal, immune and nervous system by influencing body equilibrium [5]. The authors showed that the sensitivity of thyroid follicular cells (FRTL-5 cell line) to proton irradiation was independent of their ability to communicate through connexin 32 gap junctions, but there are no indications in the literature on their possible mechanism of action. We have previously demonstrated that UV-C radiation induced apoptosis of FRTL-5 cells, by changing nuclear lipid metabolism in relation to the physiological state of cells [6]. We used FRTL-5 cells because *in vitro* they permanently express most of *in vivo* tissue-specific thyroid characteristics, such as thyroglobulin synthesis and secretion, iodide active transport, peroxidase production and thyrotropin (TSH) sensitivity [7]. It is known that TSH treatment induced thyroid cells to the proliferative state whereas TSH starvation rendered them quiescent [8]. Proliferating cells were more sensitive to UV-C radiation treatment than quiescent cells by changing phosphatidylcholine (PC) and sphingomyelin (SM) metabolism, specially in lipid localised at nuclear level. In nuclei purified from proliferating cells, irradiation stimulated neutral-sphingomyelinase (*N*-SMase) activity and inhibited sphingomyelin-synthase (SM-synthase) and phosphatidylcholine-specific phospholipase C (PC-PLC) with the consequent increase in the ceramide/diacylglycerol ratio. This effect was low in quiescent cell nuclei. The results suggested that quiescent FRTL-5 cells were more resistant to the effects of UV-C radiation because their nuclear PC and SM metabolism was less modified than that of proliferating cells [6]. In the stratosphere, depletion of ozone resulted in an enrichment of UV radiation content and global warming. It is interesting to notice that in a space environment the FRTL-5 cell changes occurred according to their physiological state, the effect being stronger in proliferating than in quiescent cells [9]. Cells did not present signs of DNA fragmentation characteristic of apoptotic process but, in contrast, showed strongly altered lipid metabolism with activation of nuclear *N*-SMase and inhibition of PC-PLC and SM-synthase [8]. The spaceflight impaired cell growth of FRTL-5 exposed to TSH; thus, the cells became similar to quiescent cells with similar SMase and SM-synthase activities. More precisely the space environment induced the cells into a pro-apoptotic state, similar to that obtained with both TSH starvation and serum withdrawal [9]. Comparing the results in different experimental models [6,9], we demonstrated that the SMase/SM-synthase ratio changed in various functional states of the cells since its value was very high in apoptotic cells, medium in pro-apoptotic cells, low in proliferating cells and very low in quiescent cells. This parameter has been suggested to be a specific marker for cell function [9]. In fact, its value is low in quiescent cells and gradually increases in proliferating, pro-apoptotic and apoptotic cells [9]. Since thyroid cells are particularly sensitive to UV-C and space environment and there are no data in the literature about the effects of protons on these cells, here we studied for the first time if the proton beams could change SM metabolism in nuclei-free lysates (NFL) and/or in purified nuclei (N) prepared from thyroid FRTL-5 at the end to identify their role on functional state of the cells.

## 2. Results and Discussion

### 2.1. Proton Beams Promote Bax Expression in FRTL-5 Cells in Relation to Their Physiological State

**Figure 1:**
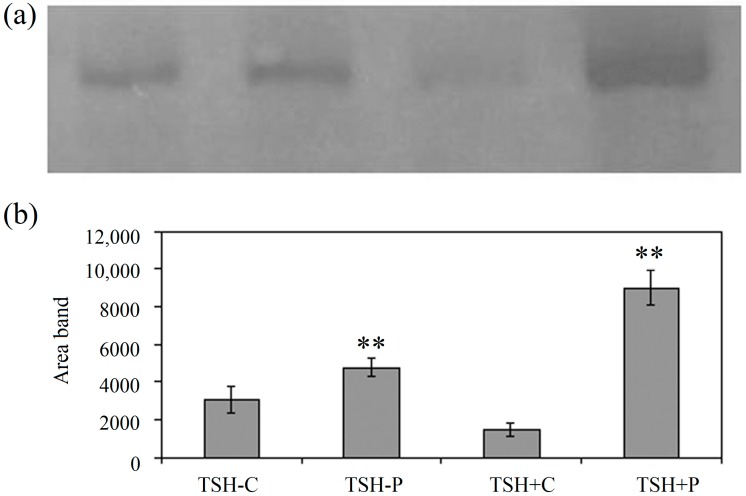
Bax analysis in quiescent control sample (TSH−C) and quiescent proton-irradiated sample (TSH−P), in proliferating control sample (TSH+C) and proliferating proton-irradiated sample (TSH+P). C, control; P, proton. (**a**) Immunoblots of proteins (30 µg) were probed with anti-Bax antibodies (apparent molecular weight 23 kDa) and visualized by ECL; (**b**) The area density was evaluated by densitometry scanning and analysed with Scion Image; data represent the means ± S.D. of four separate experiments. irradiated samples significance ** *p* < 0.01 *versus* control.

It has been demonstrated that proton beams inhibited cell growth and induced apoptosis in melanoma cells [2]. To demonstrate if the effect of protons was specific for proliferating cells, such as tumor cells or others, we used an experimental model in which the same cells can be maintained in quiescent or proliferating state. Thyroid FTRL-5 cells were cultured in the presence of glycil-l-histidyl-l-lysine acetate, hydrocortisone, insulin, somatostatin, transferrin without (TSH−) or with TSH (TSH+). As previously reported, TSH+ cells are proliferating and TSH− cells are quiescent cells [6]. Here we showed that proton beams increased expression of Bax protein specifically in proliferating cells (Figure 1). The Bax protein analysis actually showed a stronger immunopositivity in irradiated cells in comparison to control samples (Figure 1a). Bax protein levels increased approximately 1.6 and 6.2 fold, respectively in TSH− and TSH+ cells exposed to proton irradiation, compared to unirradiated quiescent and proliferating controls (Figure 1b). The changes of Bax protein levels were evaluated by the analysis of the area of immunoblotting bands as previously reported [10].

### 2.2. Proton Beams Act on Nuclear Sphingomyelin Metabolism

**Figure 2:**
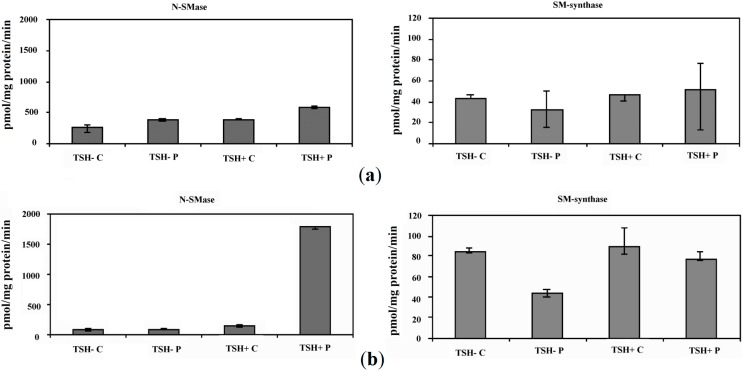
Effect of proton irradiation on neutral-sphingomyelinase (*N*-SMase) and sphingomyelin-synthase (SM-synthase) activity in: (**a**) nuclei-free lysates (NFL) and (**b**) purified nuclei (N). C, control; P, proton. Data are expressed as pmol/mg protein/min and represent the mean and range of three separated experiments.

In both NFL and N fractions *N*-SMase activity was higher in proliferating than in quiescent cells whereas SM-synthase activity did not change significantly with cell state (Figure 2a,b), supporting previous results [6]. In NFL sample, protons increased *N*-SMase activity 1.45 and 1.52 times in quiescent and proliferating cells, respectively (Figure 2a). In addition, while *N*-SMase activity increased only 1.09 times in N purified from quiescent cells, in N purified from proliferating cells its value increased 12.44 times (Figure 2b). Irradiation unchanged SM-synthase activity in NFL prepared from both cells and in N from proliferating cells whereas it was inhibited 1.9 times in N purified from quiescent cells (Figure 2a,b).

**Figure 3:**
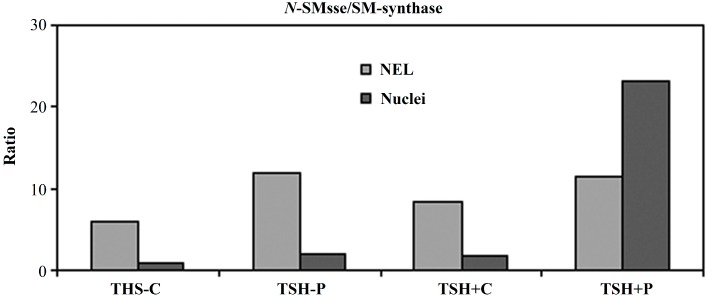
Effect of proton irradiation on neutral-sphingomyelinase/sphingomyelin-synthase (*N*-SMase/SM-synthase) ratio of nuclei-free lysates (NFL) and purified nuclei (N) prepared from quiescent (TSH−) and proliferating (TSH+) cells. C, control; P, proton.

As a consequence, *N*-SMase/SM-synthase ratio, was 8.6 in NFL and 1.77 in N prepared from proliferating cells whereas its value was 6 in NFL and 1.06 in N prepared from quiescent cells (Figure 3), similar to that previously reported [9]. The proton treatment increased the value of *N*-SMase/SM-synthase ratio to 11.75 and 8.46 in NFL and N respectively in quiescent cells and to 11.39 and 22.97 in NFL and N respectively (Figure 3). Thus, the effect of protons was stronger in nuclear fraction than in NFL.

The *N*-SMase/SM-synthase ratio has been proposed to be a specific marker for cell function [9]. Its value was 8.16 ± 1.00, 5.77 ± 0.60, 14.00 ± 0.9 and 36.73 ± 3.30 in NFL and 1.77 ± 0.2, 1.06 ± 0.10, 5.39 ± 0.8, 47.80 ± 5.2 in N prepared from proliferating, quiescent, proapoptotic and apoptotic cells respectively [8]. Therefore our results indicated that protons induced quiescent cells in a propaoptotic state as well as occurred for proliferating cells if you considered the change of *N*-SMase/SM-synthase ratio in NFL. Otherwise, if you considered *N*-SMase/SM-synthase ratio in purified N, its value was between the proapoptotic state and apoptotic state, suggesting that the metabolism of the nuclear SM was more sensitive than that of NFL and probably it was the first that changed when the cells were induced to apoptosis. It is really hard to indicate exactly why protons have more effects on the SM metabolism in N than in NFL. Since the activation of the nuclear SMase reduced the amount of SM localized in the inner nuclear membrane where binded to cholesterol (CHO) to acts as a platform for DNA duplication and transcription [11] is possible that protons use this method to induce apoptosis. These data supported the more expression of bax protein in proliferating cells than in quiescent cells treated with protons.

To study the effect of *N*-SMase/SM-synthase ratio on nuclear lipids, the most representative species of SM and ceramide in the N were studied and they were referred to CHO content. The results showed that the change of CHO observed in the samples was not statistically significant. In fact, CHO value was 9.18 ± 1.76 and 7.86 ± 2.20 ng/mg protein in TSH− and TSH+ control samples and 10.20 ± 1.2, and 11.20 ± 1.34 ng/mg protein in TSH− and TSH+ proton-treated samples.

**Figure 4:**
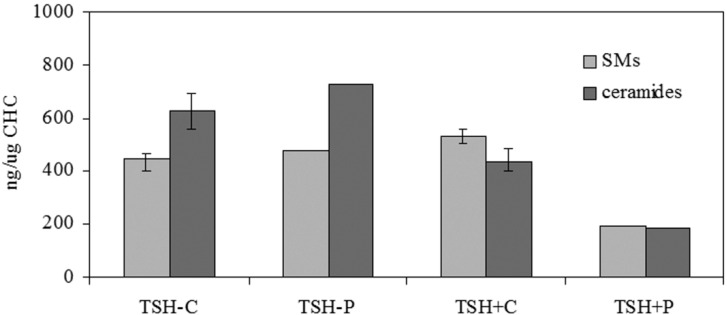
Effect of proton irradiation on total sphingomyelin (SM) and ceramide species under study of nuclei-free lysates (NFL) and purified nuclei (N) prepared from quiescent (TSH−) and proliferating (TSH+) cells. C, control; P, proton. Data are expressed as μg/μg cholesterol (CHO) and are expressed as mean and range of three separated experiments for C samples. Results of P samples are only of one experiment.

The limit of this part of the present study is that we had only one sample for the SM and ceramide analysis of proton-exposed samples and we did not have the possibility to repeat the exposure at the moment. Therefore, only strong changes were considered. The N fraction of the proliferating cells was richer in SM and poorer in ceramide content than that of quiescent cells (Figure 4). The role of nuclear SM on cell proliferation has been widely demonstrated [12,13,14]. SM facilitated DNA stabilization [12]. The trimethylammonium group of SM bonded to the phosphate group of DNA, whereas its apolar fatty acids bonded to the hydrophobic centers of the internal part of the helical DNA, facilitating its stabilization [12]. The increase of the nuclear SM concentration during cell proliferation stimulated the SMase activity during the S phase of the cell cycle, thus favoring the initiation of DNA synthesis [15]. Our results, showing the high level of SM and SMase activity in purified N of proliferating FRTL-5 cells, confirmed previous observation obtained in liver. As *N*-SMase was more active in proliferating cells, we would expect a higher content of ceramide. Instead, the content of ceramide was reduced (Figure 4), probably being rapidly moved to the cytoplasm. The most represented species were ceramide 18:1 16:0 and ceramide 18:1 24:0 and they were reduced 1.62 and 1.48 times respectively with protons (Table 1).

The proton beams treatment of quiescent cells induced a low increase of total ceramide (Figure 4) due probably to the SM-synthase inhibition more than to the low increase of *N*-SMase activity (Figure 2). In particular, ceramide 18:1 20:0 increased (Table 1). In proliferating cells treated with protons, the content of total SM was reduced 2.76 times due to the high activity of *N*-SMase (Figure 2) and the content of total ceramides was reduced 2.33 times (Figure 4). All species of ceramide were reduced with the exception of ceramide 18:1 18:0 (Table 1). We do not know at the moment if the reduction of ceramides could be due to the nucleus-cytoplasm translocation or to the presence of a ceramidase enzyme in the N fraction of FTRL-5 cells, which however has not been demonstrated until now.

**Table 1:** Species of sphingomyelin (SM) and ceramide in nuclei of FRTL-5 cells. Data of proton (P) are expressed as ng/µg cholesterol and represent the mean of two samples very similar. TSH−, quiescent cells; TSH+, proliferating cells; C, control; P, proton. Data of control (C) are expressed as mean and range of three independent experiments.

SM Species	TSH−C	TSH−P	TSH+C	TSH+P
SM 18:1 12:0	0.7 (0.5–0.8)	0,9	0.3 (0.2–0.6)	0.15
SM 18:1 16:0	46 (41–58)	50	64 (55–71)	20
SM 18:1 18:1	5 (3.3–5.6)	2,2	1.4 (1.1–1.5)	1.4
SM 24:0	172 (160–203)	184	188 (168–202)	74
ceramide 18:1 16:0	117 (102–139)	147	72 (62–68)	25
ceramide 18:1 18:0	31 (23–36)	48	2 (1.6–2.3)	8
ceramide 18:1 20:0	45 (18–54)	118	62 (40–71)	27

## 3. Experimental Section

### 3.1. Reagents and Standards

PC, SM, non-hydroxy fatty acid ceramide, phenylmethylsulfonylfluoride, acetonitrile, methanol, 2-propanol, metyl-tert-butyl ether (MTBE), formic acid, chloroform, cholesterol (CHO) were obtained from Sigma Chemical Co. (St. Louis, MO, USA.); TLC plates (silica Gel G60) were from Merck (Darmstadt, Germany); the radioactive [Me-^14^C] SM (54.5 Ci/mol, 2.04 GBq/mmol), [Me-^3^H] (l-3-phosphatidyl-[*N*-Me-^3^H]-choline-1,2-dipalmitoyl, 81.0 Ci/mmol, 3.03 TBq/mmol) were from Amersham Pharmacia Biotech (Rainham, Essex, UK); Ecoscint A was from National Diagnostic (Atlanta, GA, USA.). Anti-Bax was obtained from Santa Cruz Biotechnology, Inc. (Dallas, TX, USA). SDS-PAGE Molecular Weight Standard was from Bio-Rad Laboratories (Hercules, CA, USA). SM 18:1 12:0, SM 18:1 16:0, SM 18:1 18:1, SM 24:0, ceramide 18:1 16:0, ceramide 18:1 18:0, ceramide 18:1 20:0, ceramide 18:1 24:0 were purchased from Avanti (Avanti Polar, Alabaster, AL, USA).

### 3.2. Cell Cultures and Treatments

FRTL-5 cells were prepared and characterized in the Ambesi-Impiombato laboratory as previously reported [9]. Cells were grown in Ham’s modified F-12 with 5% calf serum and 6 hormones: 10 ng/mL glycil-l-histidyl-l-lysine acetate (Sigma), 10^−8^ M hydrocortisone (Sigma), 10 µg/mL insulin (Sigma), 10 µg/mL somatostatin (Sigma), 5 µg/mL transferrin (Sigma), 10 mU/mL TSH (Sigma). FRTL5 were maintained at 37 °C in 5% of CO_2_, 95% humidity incubator. The cells cultured in the presence of TSH that stimulates cell proliferation were called TSH+ cells, whereas the cells cultured without TSH remained in the quiescent state and were called TSH− cells [6]. Cells were counted and seeded at 5 × 10^5^/60 mm plastic dish concentration in TSH+ medium to permit adhesion. After 24 h, culture continued for 7 days in TSH+ or TSH− medium. For the experiment, proliferating and quiescent FRTL-5 cells were collected and then seeded at a density of 1 × 10^6^ cells/1.0 cm^2^ chamber with 1 mL TSH+ or TSH− medium for 24 h in incubator to permit adhesion and then each chamber placed inside boxes suitable for cultivation where the temperature was set at 37 °C. After plating, the cells were submitted to proton beams produced and accelerated by the CERN (Geneva, Switzerland) accelerators. Radio-frequency linear accelerators were used as injectors for synchrotrons and as stand-alone accelerators for the production of intense particle beams, thanks to their ability to accelerate high beam currents at high repetition rates. The cells were used in part for NFL and N preparation and in part for the analysis of Bax apoptotic protein. Both NFL, containing all cell membranes except nuclear membranes, and purified N were used to analyze the effect of proton beams on SM localized in different subcellular compartments. NFL and N were prepared as previously reported [6]. Cells were washed twice with PBS and centrifuged at 800× *g* for 10 min. The pellet was suspended in hypotonic buffer (1.5 M sucrose, 3 mM CaCl_2_, 2 mM Mg acetate, 0.5 mM dithiothreitol, 1 mM PMSF, 3 mM Tris–HCl pH 8.0, 1 mL/10^6^ cells) and gently homogenized by a tight-fitting teflon-glass homogenizer. Part of the homogenate was centrifuged at 500× *g* for 30 min at 4 °C for NFL preparation and part was used for nuclei isolation. At this end homogenized cells were treated with 1% Triton X-100 in hypotonic buffer (0.5:1 *v*/*v*); the cellular suspension was stirred on a vortex mixer for 30 s and the buffer, containing 1.5 M sucrose, was added (0.25:1 *v*/*v*). After centrifugation at 2000× *g* for 10 min the pellet containing nuclei was washed twice with Barnes *et al.* solution [16] 0.085 M KCl, 0.0085 M NaCl, 0.0025 M MgCl_2_, trichloroacetic acid–HCl 0.005 M, pH 7.2).

### 3.3. Lipid Extraction

Lipid extraction was performed according to Matyash *et al.* 2008 [17]. Pellets of NFL and N were diluted with 1 mL methanol. Exactly 3 mL ultra pure water and 3 mL MTBE were added. Each sample was vortexed for 1 min and centrifuged at 3000× *g* for 5 min. The supernatant was recovered. The extraction with MTBE was repeated on the pellet and the supernatant was added to the first. The organic phase was dried under nitrogen flow and resuspended in 500 µL of methanol.

### 3.4. Ultra Fast Liquid Chromatography Tandem Mass Spectrometry (UFLC-MS/MS)

Lipid standards (SM 18:1 12:0, SM 18:1 16:0, SM 18:1 SM 18:1, ceramide 18:1 16:0, ceramide 18:1 18:0, ceramide 18:1 20:0, ceramide 18:1 24:0; sphinganine 18:1; glucosyl ceramide 18:1 16:0 and CHO) were prepared according to Matyash *et al.* [17]. Standards were dissolved in chloroform/methanol (9:1 *v*/*v*) at 10 μg/mL final concentration. The stock solutions were stored at −20 °C. Working calibrators were prepared by diluting stock solutions with methanol to 500:0, 250:0, 100:0, 50:0 ng/mL final concentrations. Twenty micro liters of standards or lipids extracted from NRL or N samples were injected after purification with specific nylon filters (0.2 µm).

Analyses were carried out according to Rabagny *et al.* [18] by using Ultra Performance Liquid Chromatography system tandem Mass Spectometer Applaied biosistem (Shimadzu Italy s.r.l., Milano, Italy). The lipid species were separated, identified and analyzed by following the methods of Rabagny *et al.* [18].

### 3.5. Neutral-Sphingomyelinase Assay

The *N*-SMase activity was detected as previously reported [6] in NFL and in N. The reaction mixture contained 0.1 M Tris–HCl pH 7.6, 0.1 mM-^14^C SM, 6 mM MgCl_2_, 0.1% Triton X-100 and 100 μg protein of NFL or N to a final volume of 0.1 mL. Incubations were performed at 37 °C for 45 min. The reaction was stopped by adding 2 mL chloroform and methanol (2:1), 0.4 mL of 0.5% NaCl was added to the tubes and vortexed. After centrifugation at 2000 rpm × 10 min, the upper phase was removed and 0.5 mL was diluted in counting vials with 10 mL Ecoscint A and 1 mL distilled water; radioactivity was measured with a Packard liquid scintillation analyzer. Protein determination was performed as previously reported [6] and the enzyme activity was referred to protein content.

### 3.6. Sphingomyelin-Synthase Assay

The SM-synthase activity was detected as previously reported [6] in NFL and in N. The reaction mixture contained 0.1 M Tris–HCl, 0.3 mM ^3^H-PC, 2 mM CaCl_2_, 0.1% Triton X-100, 0.15 mM non-hydroxy fatty acid ceramide and 100 µg protein of NFL or N, to a final volume of 0.1 mL. Incubations were performed at 37 °C for 45 min. The reaction was stopped by adding 2 mL chloroform and methanol (2:1), 0.4 mL of 0.5% NaCl was added to the tubes and vortexed. After centrifugation at 2000 rpm × 10 min, the lower phase was dried under nitrogen flow, lipids were re-suspended with chloroform and separated with thin-layer silica gel chromatography (TLC), using chloroform/methanol/ammonia (65:25:4) as solvent. In the sample, exogenous SM was added to the tubes before chromatography. SM was localized with iodine vapor, scraped into counting vials and diluted with 10 mL Ecoscint A and 1 mL water. Radioactivity was measured as reported for *N*-SMase activity.

### 3.7. Immunoblotting Analysis

Bax protein was evaluated with western blot analysis. About 30 µg proteins were loaded onto SDS-PAGE electrophoresis in 10% polyacrylamide slab gel and transferred into nitrocellulose in 75 min as previously reported [6]. Membranes were blocked for 30 min with 0.5% no fat-dry milk in PBS, pH 7.5, and incubated over night at 4 °C with anti-Bax antibodies. Blots were treated with horseradish-conjugated secondary antibodies for 90 min. Visualization was performed with the enhanced Chemiluminescence’s kit from Amersham Pharmacia Biotech. Immunoblot bands were quantified by Scion Image program.

### 3.8. Statistical Analysis

Data represent the median and range of three separated samples for enzyme activities and lipids and the means ± S.D. of four separate experiments for Bax analysis.

## 4. Conclusions

Proton beams induced epithelial thyroid cells in a proapoptotic state if the cells were quiescent whereas in an initial apoptotic state if the cells were proliferating, by changing particularly the nuclear SM metabolism. In cell N the strong activation of SMase reduced SM content that was important for the DNA stability. The produced ceramide probably was translocated to the cytoplasm where could be metabolized to sphingosine and sphingosine-1-phosphate, mediators involved in apoptosis. Our data indicate that nuclear SM metabolism is involved in proton-induced damage.
